# Impact of *Plasmodium falciparum* gene deletions on malaria rapid diagnostic test performance

**DOI:** 10.1186/s12936-020-03460-w

**Published:** 2020-11-04

**Authors:** Michelle L. Gatton, Alisha Chaudhry, Jeff Glenn, Scott Wilson, Yong Ah, Amy Kong, Rosalynn L. Ord, Roxanne R. Rees-Channer, Peter Chiodini, Sandra Incardona, Qin Cheng, Michael Aidoo, Jane Cunningham

**Affiliations:** 1 Queensland University of Technology, Brisbane, QLD Australia; 2 The CDC Foundation, Atlanta, GA USA; 3 Centers for Disease Control and Prevention, Atlanta, USA; 4 Hospital for Tropical Diseases, London, UK; 5 London School of Hygiene and Tropical Medicine, London, UK; 6 Foundation for Innovative New Diagnostics (FIND), Geneva, Switzerland; 7 Australian Defence Force Malaria and Infectious Diseases Institute (FORMERLY Australian Army Malaria Institute), Brisbane, QLD Australia; 8 World Health Organization, Geneva, Switzerland

**Keywords:** Rapid diagnostic tests, Hisidine rich protein 2, HRP2, Gene deletion

## Abstract

**Background:**

Malaria rapid diagnostic tests (RDTs) have greatly improved access to diagnosis in endemic countries. Most RDTs detect *Plasmodium falciparum* histidine-rich protein 2 (HRP2), but their sensitivity is seriously threatened by the emergence of *pfhrp2*-deleted parasites. RDTs detecting *P. falciparum* or pan-lactate dehydrogenase (Pf- or pan-LDH) provide alternatives. The objective of this study was to systematically assess the performance of malaria RDTs against well-characterized *pfhrp2*-deleted *P. falciparum* parasites.

**Methods:**

Thirty-two RDTs were tested against 100 wild-type clinical isolates (200 parasites/µL), and 40 samples from 10 culture-adapted and clinical isolates of *pfhrp2*-deleted parasites. Wild-type and *pfhrp2*-deleted parasites had comparable Pf-LDH concentrations. Pf-LDH-detecting RDTs were also tested against 18 clinical isolates at higher density (2,000 parasites/µL) lacking both *pfhrp2* and *pfhrp3.*

**Results:**

RDT positivity against *pfhrp2*-deleted parasites was highest (> 94%) for the two pan-LDH-only RDTs. The positivity rate for the nine Pf-LDH-detecting RDTs varied widely, with similar median positivity between double-deleted (*pfhrp2/3* negative; 63.9%) and single-deleted (*pfhrp2*-negative/*pfhrp3*-positive; 59.1%) parasites, both lower than against wild-type *P. falciparum* (93.8%). Median positivity for HRP2-detecting RDTs against 22 single-deleted parasites was 69.9 and 35.2% for HRP2-only and HRP2-combination RDTs, respectively, compared to 96.0 and 92.5% for wild-type parasites. Eight of nine Pf-LDH RDTs detected all clinical, double-deleted samples at 2,000 parasites/µL.

**Conclusions:**

The pan-LDH-only RDTs evaluated performed well. Performance of Pf-LDH-detecting RDTs against wild-type *P. falciparum* does not necessarily predict performance against *pfhrp2*-deleted parasites. Furthermore, many, but not all HRP2-based RDTs, detect *pfhrp2*-negative/*pfhrp3*-positive samples, with implications for the HRP2-based RDT screening approach for detection and surveillance of HRP2-negative parasites.

## Background

Antigen-detecting rapid diagnostic tests (RDTs) are recommended diagnostic tools by the World Health Organization (WHO) for malaria case management [1]. The implementation of malaria RDTs has greatly improved access to diagnosis in endemic countries, particularly in Africa [2].

In general, three types of RDTs for detection of *Plasmodium falciparum* are commercially available: 1) *P. falciparum*-only RDTs; 2) combination RDTs, which detect and differentiate *P. falciparum* and some, or all, non-*P. falciparum* species; and, 3) Pan-only RDTs, which detect but do not differentiate between *P. falciparum* and non-*P. falciparum* species. Most *P. falciparum*-detecting RDTs use histidine-rich protein 2 (HRP2) as it is species-specific and abdundantly produced. Some HRP2-based RDTs may also potentially detect *P. falciparum* histidine-rich protein 3 (HRP3) due to its structural similarity with HRP2 [3]. RDT bands detecting non-falciparum *Plasmodium* target Pan or species-specific lactate dehydrogenase (Pan-LDH, *Plasmodium vivax* (Pv)-LDH or *P. vivax, Plasmodium ovale, Plasmodium malariae* (Pvom)-LDH), or aldolase. *P. falciparum*-LDH (Pf-LDH) can also be used specifically to detect *P. falciparum*. HRP2-based RDTs generally exhibit superior performance, particularly at low parasite densities, and are more heat stable than non-HRP2-based RDTs [4].

The sensitivity of HRP2-based RDTs is seriously threatened by the increasing occurrence of *P. falciparum* with deleted HRP2 and/or HRP3 antigen-coding genes. *Plasmodium falciparum* isolates lacking *pfhrp2/3* were first reported in Peru with a prevalence of 20 to 90%, depending on location [5–7]. Subsequently, *pfhrp2/3*-deleted parasites have been reported in Colombia, Suriname, Bolivia, Brazil, Honduras, Guatemala, and Nicaragua [8–12]. Parasites lacking *pfhrp2* have also been reported around the China-Myanmar border [13] and in India [14] with prevalence up to 25% in some areas [15].

In Africa, parasites lacking one or both *pfhrp2* and *pfhrp3* have been reported in Mali [16], Ghana [17], Senegal [18], Democratic Republic of the Congo [19], Rwanda [20], Zambia [21], and Kenya [22] with prevalences ranging between 2 and 45%, while up to 80% of symptomatic patients at two regional hospitals in Eritrea had *P. falciparum* lacking *pfhrp2/3* [23].

The emergence of parasites that do not express HRP2 poses a major public health threat due to the heavy reliance of RDTs on this antigen. In response, WHO has released a Response Plan [24]. If HRP2-based *P. falciparum*-only RDTs are used when a patient is infected solely with parasites lacking HRP2 then a false-negative diagnosis can occur, delaying correct treatment and potentially leading to severe complications and death. In regions where HRP2-pan-LDH combination tests are used, incorrect diagnosis of non-falciparum malaria can occur when individuals are infected with HRP2-negative *P. falciparum*, potentially impacting the treatment regimen and patient health outcome. In both situations, routine surveillance estimates of malaria incidence will be adversely affected.

The current solution to the diagnostic problem posed by *P. falciparum* parasites lacking *pfhrp2/3* is first to establish prevalence and, based on these results, decide if a replacement RDT or microsopy is needed. Any replacement RDT should not exclusively rely on HRP2 for *P. falciparum.* However, it can be challenging to maintain access to quality-assured microscopy and to switch RDTs, especially as the number of RDTs targeting alternative antigens is limited. Eighty-nine RDTs that detect *P. falciparum,* alone or in combination, have undergone WHO product testing within the past 5 years [4]. Of these, 78 use HRP2 exclusively to detect *P. falciparum*, with nine currently pre-qualified by WHO. The remaining 11 RDTs use Pf-LDH either alone or in combination with HRP2, or pan-LDH only, to detect *P. falciparum*, with only two of these products (one pan-LDH and one HRP2/Pf-LDH) being WHO pre-qualified [4]. During WHO product testing, the performance of Pf-LDH test bands has been generally poor with only two products having met the WHO performance criteria based on Pf-LDH test line results against wild-type, HRP2-expressing *P. falciparum*.

Although many studies have evaluated RDT performance in the field, and the WHO and Foundation for Innovative New Diagnostics, in collaboration with the US Centers for Disease Control and Prevention (CDC), have led rigorous performance testing of RDTs over the past decade, there has been no systematic assessment of the performance of RDTs against well-characterized *pfhrp2*-deleted *P. falciparum* parasites.

To address this gap, Round 8 WHO Malaria RDT Product Testing Programme included an evaluation of RDTs against a panel of *pfhrp2*-deleted *P. falciparum* parasites, with and without *pfhrp3*. Here, the findings are described and discussed with reference to the implications for future RDT use.

## Methods

### Parasite samples

Two panels of *P. falciparum* were tested against all RDTs in this study: 1) wild-type panel of 100 clinical *pfhrp2*-positive isolates, and 2) *pfhrp2*-deleted panel containing 40 samples from 10 different isolates/strains (Table 1). All samples were genotyped for *pfhrp2* and *pfhrp3* as previously described [6]. All seven Loreto clinical isolates in the *pfhrp2*-deleted panel and parasites from the 3BD5 culture line were confirmed to be negative for both *pfhrp2* and *pfhrp3*, while the D10 and Dd2 parasites were confirmed to be *pfhrp2*-negative/*pfhrp3*-positive.

**Table 1:** Characteristics of parasite panels used to test malaria rapid diagnostic tests

Panel	No. samples	Characteristics	Antigen concentration (ng/mL)		RDTs tested
			HRP2	Pf-LDH	
Wild-type	100	Panel composition: 100 diluted clinical samples each at 200 parasites/µL *pfhrp2* status: confirmed *pfhrp2*-positive Sample origins: Central African Republic (n = 1), Colombia (n = 6), Ethiopia (n = 1), Kenya (n = 1), Cambodia (n = 17), Myanmar (n = 1), Nigeria (n = 51), Peru (n = 6), Philippines (n = 1), Senegal (n = 5) and Tanzania (n = 10)	Mean: 11.76; Median: 6.76; Range: 0.67-62.48	Mean: 16.13; Median: 13.59; Range: 0.19-53.53	All
*pfhrp2*-deleted	40	Panel composition: 7 diluted clinical samples (200 parasites/µL) from Loreto region of Peru plus 33 culture-adapted samples (11 serial dilutions x Dd2, D10 and 3BD5) with Pf-LDH concentrations equivalent to 200 parasites/µL; *pfhrp2* status: 18 samples confirmed to be *pfhrp2/3*-deleted (double-deleted; 7 clinical samples plus 11 dilutions of 3BD5 strain); 22 samples confirmed to be *pfhrp2*-deleted with intact *pfhrp3* (single-deleted; 11 dilutions each of Dd2 and D10 strains)	Mean: 0.27; Median: 0.11; Range: 0.00–1.70; Range (single-deleted): 0.10-1.70; Range (double-deleted): 0.00-0.20	Mean: 13.75; Median: 9.85; Range: 2.50–58.00	All
Double-deleted (higher density)	18	Panel composition: 7 diluted clinical samples (2000 parasites/µL) from Loreto region of Peru plus 11 samples of culture-adapted 3BD5 with tenfold higher Pf-LDH concentrations than used in *pfhrp2*-deleted panel *pfhrp2* status: confirmed *pfhrp2/3*-deleted	Mean^a^: 0.07; Median^a^: 0.00 Range^a^: 0.00–0.37	Mean^a^: 224.25; Median^a^: 193.78; Range^a^: 47.50-526.00	RDTs with Pf-LDH test band

*RDT* rapid diagnostic test, *HRP2* histidine-rich protein 2, *Pf-LDH P. falciparum* lactate dehydrogenase

^a^Values calculated for clinical samples only. Antigen concentrations were not determined for higher density culture samples because the levels were measured for the same samples at a 1:10 dilution for the *pfhrp2*-deleted panel

The three culture-adapted parasites lines were grown to between 1 and 2% parasitaemia using standard culture techniques [25], harvested and frozen at –70 °C. After determination of antigen (HRP2, Pf-LDH and aldolase) concentration by ELISA in the stock parasite preparations (methods below), frozen parasites were diluted using PCR-confirmed malaria negative group O blood. Eleven dilutions of each culture strain were generated with Pf-LDH concentration distributions similar to those in the wild-type panel (Table 1). A higher priority was given to matching the Pf-LDH distribution between panels because of the dominance of RDTs using this antigen, compared to aldolase (only one Round 8 product targeted aldolase).

A supplemental panel of double-deleted clinical and culture parasites at higher density was also produced using high density stocks of the same double-deleted parasite samples as in the *pfhrp2*-deleted panel, using the methods above (Table 1). This panel consisted of the seven clinical isolates from Peru, diluted to 2,000 parasites/µL [26], and the 11 samples of the 3DB5 strain at tenfold higher concentration than used in the *pfhrp2*-deleted panel described above.

### Measurement of antigen concentrations

HRP2 and Pf-LDH concentrations in the stock and diluted samples of the *pfhrp2*-deleted panel were measured by commercial ELISA following manufacturers’ instructions; Malaria Antigen Cellisa kit (Celllabs PTY LTD, Brookvale, NSW, Australia) for HRP2 and Qualisa malaria antigen pLDH ELISA kit (Tulip Diagnostics Ltd, Alto Santacruz, Goa, India) for *Plasmodium*-pLDH. Antigen concentrations of HRP2 and Pf-LDH were determined based on a standard curve run on each plate produced from serially diluted recombinant HRP2 and Pf-LDH antigens, respectively. In addition, samples with known concentrations of antigen were run as internal controls for the assay.

The aldolase determinations were done using an in-house ELISA. Capture (M/B 7-20) and detection antibodies (mAb C/D 11-4) were obtained from the National Bioproducts Institute (Pinetown, South Africa). The detection antibody mAb C/D 11-4 was biotinylated using EZ-Link Sulfo-NHS-Biotin (ThermoFisher Scientific, Waltham, MA, USA). Recombinant *P. falciparum* aldolase antigen (Microcoat Biotechnologie GmbH, Germany), diluted in human malaria-negative blood was used to generate a standard curve from which aldolase concentrations were determined. For all ELISAs, each sample was run in duplicate, three or more times, on consecutive days and the antigen concentration determined based on the average of three runs.

### RDT testing procedure

Each RDT product was tested against the wild-type and *pfhrp2*-deleted panels, with each sample tested in duplicate on two product lots by trained technicians blind to the randomized sample order. RDT band intensities were noted using a colour intensity chart as per the RDT Product Testing Standard Operating Procedures and results were double-entered into the WHO Product Testing database [26].

The RDT products targeting Pf-LDH were also tested against the double-deleted (higher-density) parasite panel. This testing was only conducted on one product lot, independent of testing the low-density wild-type and *pfhrp2*-deleted panels.

### RDT characteristics and categorization

Thirty-two RDTs from 17 manufacturers were assessed. These were a sub-set of the 34 RDTs tested during Round 8 of the WHO Malaria RDT Product Testing Programme [4]; only RDTs with a false-positive rate below 10% against parasite-negative samples were included in the current study. The included RDTs are listed in four groups in Table 2 according to the target antigens detected and the a priori expected detection of *pfhrp2*-deleted parasites:

**Table 2:** Malaria RDT products included in evaluation

Manufacturer	Product name	Product code	Target antigen(s)
Pf-LDH detection RDTs (Group 1)			
Access Bio, Inc.	CareStart™ Malaria Pf (HRP2/pLDH) Ag Combo 3-line RDT	RMSM-02571	Pf-LDH, HRP2
Access Bio, Inc.	CareStart™ Malaria Pf (HRP2/pLDH) Ag RDT	RMPM-02571	Pf-LDH/HRP2
Access Bio, Inc.	CareStart™ Malaria Pf/PAN (pLDH) Ag RDT	RMLM-02571	Pan-LDH, Pf-LDH
Access Bio Ethiopia	CareStart™ Malaria Pf (HRP2/pLDH) Ag RDT	RMPM-02591	Pf-LDH/HRP2
WELLS BIO, INC	careUS^TM^ Malaria Combo Pf (HRP2/pLDH) Ag	RMP-M02582	Pf-LDH/HRP2
Advy Chemical Pvt. Ltd.	EzDx Malaria Pf Rapid malaria Antigen detection test (pLDH)	RK MAL 024-25	Pf-LDH
Meril Diagnostics Pvt Ltd.	MERISCREEN Malaria pLDH Ag	MVLRPD-02	Pan-LDH, Pf-LDH
Standard Diagnostics Inc. (Alere)	SD BIOLINE Malaria Ag P.f (HRP2/pLDH)	05FK90	Pf-LDH, HRP2
Standard Diagnostics Inc. (Alere)	SD BIOLINE Malaria Ag P.f/P.f/P.v	05FK120	Pf-LDH, Pv-LDH, HRP2
Pan-LDH only (Group 2)			
Access Bio Ethiopia	CareStart™Malaria PAN (pLDH) Ag RDT	RMNM-02591	Pan-LDH
WELLS BIO, INC	careUS^TM^ Malaria PAN (pLDH) Ag	RMN-M02582	Pan-LDH
HRP2-only (Group 3)			
Access Bio, Inc.	CareStart™ Malaria Pf (HRP2) Ag RDT	RMOM-02571	HRP2
Orchid Biomedical Systems (Tulip Group)	Paracheck Pf^®^ Rapid Test for Pf Malaria (Ver. 3)	302030025	HRP2
SD Biosensor	STANDARD Q Malaria P.f Ag Test	09MAL10B	HRP2
Omega Diagnostics Ltd.	VISITECT^®^ Malaria Pf	OD336	HRP2
HRP2-combination (Group 4)			
ASPEN Laboratories PVT.LTD	Aspen^®^ Mal (Ag Pf/Pv) Rapid Card Test	AS1550E	Pv-LDH, HRP2
Access Bio, Inc.	CareStart™ Malaria Pf/PAN (HRP2/pLDH) Ag Combo RDT	RMRM-02571	Pan-LDH, HRP2
Access Bio, Inc.	CareStart™ Malaria Pf/VOM (HRP2/pLDH) Ag Combo RDT	RMWM-02571	Pvom-LDH, HRP2
Access Bio, Inc.	CareStart™ Malaria Pf/Pv (HRP2/pLDH) Ag Combo RDT	RMVM-02571	Pv-LDH, HRP2
Access Bio Ethiopia	CareStart™Malaria Pf/PAN (HRP2/pLDH) Ag Combo RDT	RMRM-02591	Pan-LDH, HRP2
WELLS BIO, INC	careUS^TM^ Malaria Combo Pf/PAN (HRP2/pLDH) Ag	RMR-M02582	Pan-LDH, HRP2
Assure Tech (Hangzhou)	Ecotest Malaria P.f/Pan Rapid Test Device	MAL-W23M	Pan-LDH-HRP2
Nantong Egens Biotechnology Co., Ltd.	EGENS Malaria Pv/Pf Test Cassette	MAL-W23M (p.f/p.v)	Pv-LDH, HRP2
Zephyr Biomedicals	FalciVax™ Rapid Test for Malaria Pv/Pf	503010025	Pv-LDH, HRP2
Premier Medical Corporation Private Ltd.	First Response^®^ Malaria Ag. P.f./P.v. Card test^c^	PI19FRC25	Pv-LDH, HRP2
Karwa Enterprises pvt ltd	Karwa^®^ Mal (Ag Pf/Pv) Rapid Card Test	KW 1550E	Pv-LDH, HRP2
Hangzhou AllTest Biotech Co. Ltd.	Malaria P.f./Pan Rapid Test Cassette	IMPN-402	pan-aldolase, HRP2
Meril Diagnostics Pvt Ltd.	MERISCREEN Malaria Pf/Pan Ag	MHLRPD-02	Pan-LDH, HRP2
Nectar Lifesciences Limited	Necviparum One Step Malaria P.f./P.v. Antigen Test	MAGDR	Pv-LDH, HRP2
Zephyr Biomedicals	Parascreen^®^ Rapid Test for Malaria Pan/Pf	503030025	Pan-LDH, HRP2
SD Biosensor	STANDARD Q Malaria P.f/Pan Ag Test	09MAL30B	Pan-LDH, HRP2
SD Biosensor	STANDARD Q Malaria P.f/P.v Ag Test	09MAL20B	Pv-LDH, HRP2

Target antigens captured by each test band are separated by a comma; target antigens captured on the same test band are indicated using a forward slash (/). HRP2: histidine-rich protein 2; Pf-LDH: *P. falciparum* lactate dehydrogenase; Pan-LDH: pan lactate dehydrogenase; Pv-LDH: *P. vivax* lactate dehydrogenase; Pvom-LDH: *P, vivax, ovale* and *malariae* lactate dehydrogenase

1. Group 1 RDTs detecting *P. falciparum* using Pf-LDH alone or in combination with other antigens (n = 9); expected to detect and correctly identify *pfhrp2*-deleted *P. falciparum*.
2. Group 2 RDTs that detect *P. falciparum* using pan-LDH alone (n = 2); expected to detect *pfhrp2*-deleted *P. falciparum* as a *Plasmodium* positive sample.
3. Group 3 RDTs that detect *P. falciparum* only using HRP2 (n = 4); expected to return false-negative results against *pfhrp2*-deleted *P. falciparum* samples.
4. Group 4 Combination RDTs that detect *P. falciparum* using HRP2-only and other *Plasmodium* spp using pan or species-specific LDH, or aldolase (n = 17); expected to return false-negative results for falciparum infection against *pfhrp2*-deleted *P. falciparum* samples but false-positive results for non-falciparum infection (pan band positive, *P. falciparum*-specific band negative) when pan-LDH is used for one of the test lines.

Of the nine Group 1 (Pf-LDH) RDTs, three were dual-band products with separate test bands detecting Pf-LDH and HRP2, and six were single-band products using either Pf-LDH alone, or a combination of Pf-LDH and HRP2 on the same band.

RDT positivity rate was defined as the percentage of valid tests that returned a positive result on the test band for *P. falciparum* (Pf band) or a positive result for *Plasmodium* in pan-only RDTs. The RDT positivity rate is equivalent to (100–false-negative rate). Valid tests were those which returned a positive control band. Since all samples were PCR-confirmed as *P. falciparum* only, any positive *P. vivax* or Pvom test line, or any positive pan test line in the absence of a positive Pf band, represents a false positive for non-falciparum infection. The non-falciparum false positivity rate was the percentage of tests that returned a false-positive result for non-falciparum infection. It was not possible to determine non-falciparum false-positivity rates for pan-only (Group 2) and Pf-only (Group 3) RDTs because they do not differentiate species or have the capacity to detect non-falciparum infections, respectively.

### Statistical analysis

This study reports descriptive statistics only. No formal statistical testing was conducted due to the small number of RDTs in each RDT group and the small number of samples within the *pfhrp2*-deleted panel, especially when separated into single and double-deleted samples.

## Results

### RDT positivity rates against *pfhrp2*-deleted panel compared to wild-type panel

**Fig. 1:**
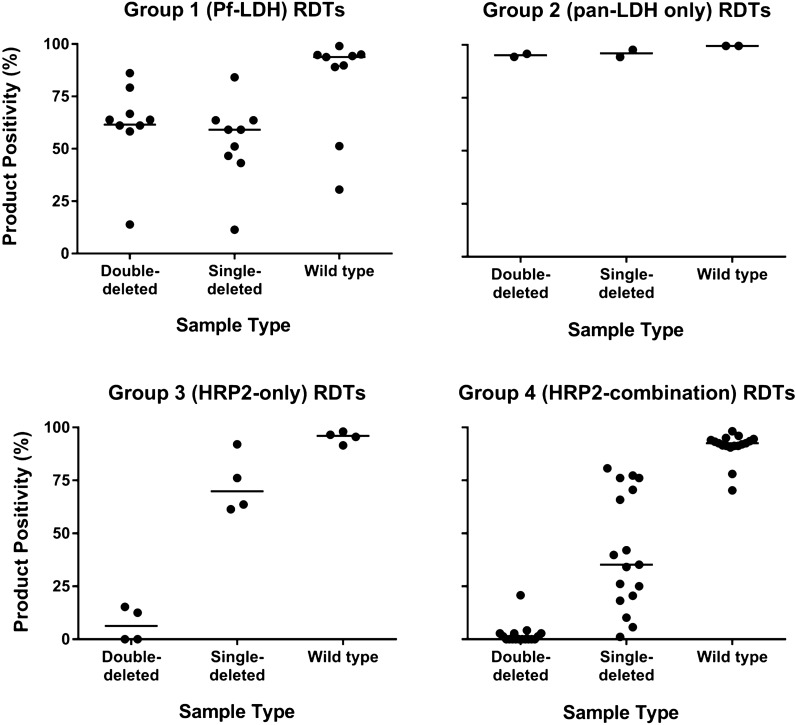
RDT positivity for individual products according to RDT group and sample type Lines indicate the median positivity for each RDT group against each sample type

The overall positivity of RDTs against *pfhrp2*-deleted parasites was 40.1%, differing by RDT group: 57.1% for Group 1 (Pf-LDH), 95.6% for Group 2 (pan-LDH only), 43.4% for Group 3 (HRP2-only), and 23.7% for Group 4 (HRP2-combination). The positivity rates were lower than against the wild-type panel, especially for Group 3 (HRP2-only) and Group 4 (HRP2-combination) RDTs (Table 3). There was wide variability in the positivity of Group 1 (Pf-LDH) RDTs, with product-specific positivity being similar between the double and single-deleted parasites, but lower than wild-type parasites (Table 3, Fig. 1). Large differences in positivity were observed between the double-deleted, single-deleted and wild-type parasites for Group 3 (HRP2-only) and 4 (HRP2-combination) RDTs, and Group 4 RDTs also showed large inter-product variation in Pf band positivity against single-deleted *P. falciparum* parasites (Table 3, Fig. 1). The positivity of individual products and lots are available from the Round 8 WHO malaria RDT product testing report [4].

**Table 3:** RDT Pf band positivity against *pfhrp2*-deleted and wild type panels according to RDT group

RDT Group	Median positivity^a^ (%) (min–max)		
	*Pfhrp2*-deleted panel		Wild type panel (n = 400^b^)
	Double-deleted (n = 72^b^)	Single-deleted (n = 88^b^)	
1 (Pf-LDH) (n = 9)	63.9 (13.9–86.1)	59.1 (11.4–84.1)	93.8 (30.5–99.0)
2 (pan-LDH only) (n = 2)	95.1 (94.4–95.8)	96.0 (94.3–97.7)	99.5 (99.5–99.5)
3 (HRP2-only) (n = 4)	6.3 (0.0–15.3)	69.9 (61.4–92.0)	96.0 (91.5–98.0)
4 (HRP2-combination) (n = 17)	0.0 (0.0–20.8)	35.2 (1.1–80.7)	92.5 (70.3–98.2)

For Group 2 (pan-LDH only) RDTs the pan band is used to determine positivity

^**a**^Positivity was calculated for each RDT against the relevant parasite panel; median, minimum and maximum positivity were then calculated across the RDTs within each group, respectively. Only valid RDT results are included in positivity calculations

^b^Each sample in each panel was tested against four RDTs of the same product

Three Group 1 (Pf-LDH) RDTs had a separate HRP2-detecting test band, in addition to the Pf-LDH test band. The positivity of this HRP2 band ranged from 0.0 to 1.4% against double-deleted parasites and 0.0 to 12.5% against single-deleted *P. falciparum*.

### *Plasmodium falciparum* test band intensity

**Fig. 2:**
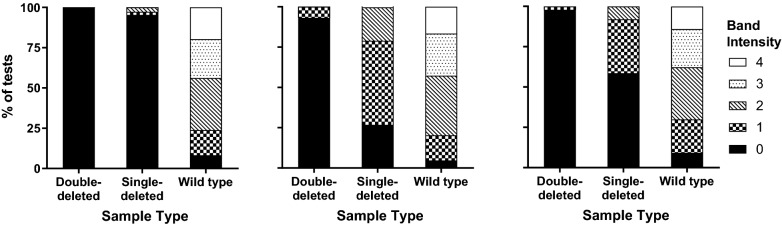
Distribution of HRP2 test band intensities according to sample type for Group 1 (Pf-LDH) RDTs which contained an independent HRP2 band (n = 3) (left), Group 3 (HRP2-only) RDTs (n = 4) (center) and Group 4 (HRP2-combination) RDTs (n = 17) (right)

**Fig. 3:**
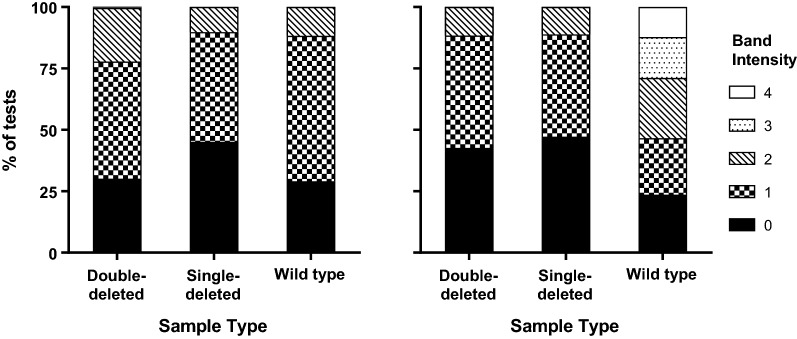
Distribution of Pf-LDH test band intensity for Group 1 (Pf-LDH) RDTs according to sample type: RDTs using both HRP2 and Pf-LDH as independent test bands to detect *P. falciparum* (n = 3) (left); RDTs only using Pf-LDH or a combined HRP2/Pf-LDH test band to detect *P. falciparum* (n = 6) (right)

Where positive results on the Pf bands of RDTs were obtained against the *pfhrp2*-deleted panel, the band intensities were weak (Figs. 2 and 3). Considering the HRP2-detecting bands only, Group 3 (HRP2-only) and Group 4 (HRP2-combination) RDTs had a higher positivity (band intensity > 0) against single deleted parasites than did the Group 1 (Pf-LDH) RDTs (Fig. 2).

There was some evidence of differences in band intensities for the Pf-LDH bands of the Group 1 (Pf-LDH) RDTs between products that used Pf-LDH alone and those that also contained an independent HRP2 test line (Fig. 3), however the number of RDTs was too small for statistical testing. The most noticeable difference was for the six RDTs that detected *P. falciparum* using Pf-LDH alone, or on a combined Pf-LDH/HRP2 test line, where there was a lower band intensity against the double- and single-deleted parasites in the *pfhrp2*-deleted panel compared to the wild-type panel (Fig. 3, right panel). In contrast, none of the three RDTs that contained independent HRP2 and Pf-LDH test bands achieved an intensity on the Pf-LDH test band above 2 on any parasite panel (Fig. 3, left panel).

### False positives for non-falciparum infection

The non-falciparum false positivity rates for Group 4 (HRP2-combination) RDTs were elevated for some products against the *pfhrp2*-deleted panel, compared to the wild-type panel, as evidenced by the large difference in maximum false-positivity rates between the different sample types (Table 4). A similar pattern was observed with two of the three combination Group 1 (Pf-LDH) RDTs, with one product returning a non-falciparum false-positive rate of 55.6% against the double-deleted *P. falciparum* parasites (Table 4). Details of non-falciparum false positives for individual products can be found in the Round 8 WHO malaria RDT product testing report [4].

**Table 4:** Non-falciparum false positivity rates for combination RDTs in Groups 1 (Pf-LDH) and 4 (HRP2-combination)

RDT Group^a^	Median non-falciparum false positivity rate^b^ (%) (min–max)		
	Double-deleted samples (n = 72)	Single-deleted samples (n = 88)	Wild type samples (n = 400)
1 (Pf-LDH) (n = 3)	13.9 (0-55.6)	21.6 (0-34.1)	2.0 (0-10.3)
4 (HRP2-combination) (n = 17)	1.4 (0-81.9)	1.1 (0-43.2)	0.8 (0 – 3.0)

^a^RDT Groups 2 (pan-LDH only) and 3 (HRP2-only) are not included as it was not possible to determine non-falciparum false positivity rates because these RDTs do not differentiate species or have the capacity to detect non-falciparum infections, respectively

^b^Non-falciparum false positivity rate was calculated for each RDT against the relevant parasite panel; median, minimum and maximum values were then calculated across the RDTs within each group. Only valid RDT results are included in non-falciparum false positivity rate calculations

### Performance of Pf-LDH RDTs against double-deleted clinical and culture samples at higher antigen concentrations

**Fig. 4:**
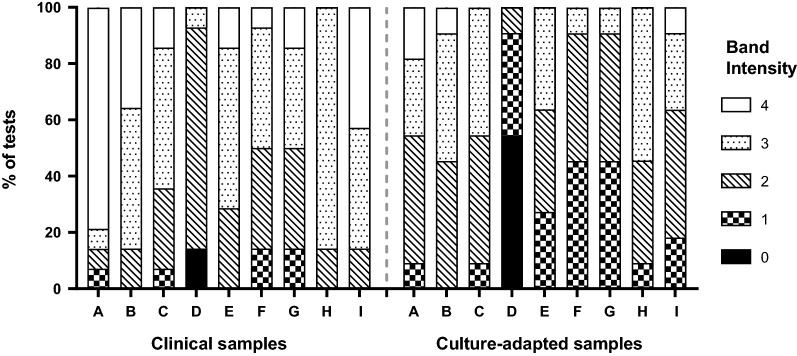
Distribution of Pf-LDH band intensities of the nine Group 1 (Pf-LDH) RDTs, labelled A to I, when tested against the double-deleted (higher density) panel containing seven clinical samples (left) and 11 culture-adapted samples (right)

The performance of the Group 1 (Pf-LDH) RDTs was assessed against the double-deleted (higher density) panel. Eight RDTs had a positivity of 100% against the seven clinical double-deleted samples, while one RDT (Product D) returned positive results on six of the seven (85.7%) samples. The mean band intensity against these clinical samples was 2.8 with some products showing a high proportion of strong (3 or 4) band intensities (Fig. 4). One of the three combination Group 1 RDTs returned one non-falciparum false-positive result (14.3%) on the pan-LDH band. The band intensity of positive tests was higher against the higher density double-deleted clinical isolates compared to the same samples at lower density (Table 5). Similar results were obtained against the higher density culture panel; eight RDTs had a positivity of 100%, while one (Product D) only detected 45% of the samples (Fig. 4).

**Table 5:** Pf-LDH band intensities for positive RDTs in Group 1 (Pf-LDH) against double-deleted clinical samples

Product	200 parasites/µl (n = 28)		2000 parasites/µl (n = 14)	
	Mean	Median (min–max)	Mean	Median (min–max)
A	1.45	1.0 (1-3)	3.57	4.0 (1-4)
B	1.52	2.0 (1-2)	3.21	3.0 (2-4)
C	1.50	1.5 (1-2)	2.71	3.0 (1-4)
D	1.00	1.0 (1-1)	2.08	2.0 (2-3)
E	1.33	1.0 (1-2)	2.86	3.0 (2-4)
F	1.17	1.0 (1-2)	2.43	2.5 (1-4)
G	1.17	1.0 (1-2)	2.50	2.5 (1-4)
H	1.52	2.0 (1-2)	2.86	3.0 (2-3)
I	1.55	2.0 (1-2)	3.29	3.0 (2-4)

Positive RDTs are those with a Pf-LDH band intensity of at least 1 on the colour intensity chart included in the RDT Product Testing Standard Operating Procedures

## Discussion

The recent emergence of *pfhrp2/3*-deleted parasites in several African and South American countries, as well as India, has rapidly escalated the need for RDTs that are not solely reliant on HRP2 for the detection of *P. falciparum*. Modelling studies have shown that use of RDTs reliant only on HRP2 detection can exert selective pressure on the parasite population to drive the spread of *pfhrp2/3*-deleted *P. falciparum* [27, 28]. The WHO recommends that countries do not exclusively rely on HRP2-based RDTs where the prevalence of *pfhrp2* deletions causing false-negative RDTs is greater than 5% in symptomatic patients [29]. In many cases this would be operationalized by changing from a HRP2-detecting RDT to a pan-LDH and/or Pf-LDH-detecting RDT, with the assumption that these RDTs perform equally well on HPR2-negative and HRP2-positive parasites. However, this assumption had not been previously tested and the current results suggest that performance of Pf-LDH-detecting RDTs against wild-type samples do not predict performance against *pfhrp2/3*-deleted parasites (clinical and cultured samples).

There was large variability in the positivity of the nine Pf-LDH RDTs tested against samples equivalent to 200 parasites/µL, but as a group they unexpectedly appeared to detect wild-type *P. falciparum* at higher rates than *pfhrp2*-deleted parasites. Indeed, one combination Pf-LDH RDT assessed in this study met the WHO performance criteria against wild-type *P. falciparum* with a Panel Detection Score (PDS) of 83 (89% positivity), but obtained a PDS of 0 (12% positivity) when assessed against *pfhrp2*-deleted parasites [4]. Antigen concentration is a potential confounder in the comparison between performance against wild-type and *pfhrp2*-deleted parasites, so the *pfhrp2*-deleted panel was prepared to have a similar distribution of Pf-LDH concentration to the wild-type panel, with all ELISAs run in triplicate using the same controls for each panel. Indeed, if the decreased positivity were due to variation in Pf-LDH concentration, it would be expected that the pan-LDH RDTs would show comparable decreases in performance when challenged against the *pfhrp2*-deleted parasites, which was not the case. Therefore, it is unlikely that differences in antigen concentrations explain the observed results.

The products using Pf-LDH included both dual-band products, with separate test bands detecting Pf-LDH and HRP2, and single-band products, using either Pf-LDH alone or a combination of Pf-LDH and HRP2 on the same band. Interestingly, the reduced performance on the *pfhrp2*-deleted panel compared to wild-type panel appeared to be restricted to Pf-LDH detecting products that did not contain a separate HRP2 band. This may be a spurious result due to the small number of Pf-LDH-detecting RDTs examined, or the limited size and diversity of the *pfhrp2*-deleted panel or product specific issues, such as Pf-LDH test lines unexpectedly reacting with HRP2. Reassuringly, all Pf-LDH RDTs were able to detect a small set of double-deleted clinical isolates at the higher parasite density of 2,000 parasites/µL.

Although assessments against larger, more geographically diverse panels are needed, these results suggest that where good quality microscopy is not available and where the prevalence of *pfhrp2/3*-deleted parasites leading to false-negative RDT results is > 5% [29], the pan-LDH RDTs would be suitable for *P. falciparum* detection. The two pan-LDH-only products had the best performance against *pfhrp2*-deleted parasites and well exceeded the minimum WHO RDT performance criteria for *P. falciparum*, specifically > 75% panel detection score at 200 parasites/µL. However, neither of these products is yet WHO pre-qualified, nor are they in the assessment pipeline, and this may limit procurement by certain agencies [30]. The one pan-LDH-only RDT that is currently WHO pre-qualified has not been evaluated against a *pfhrp2*-deleted panel and these current results highlight the need for additional assessments.

On the other hand, in areas that require differentiation of *P. falciparum* from non-*P. falciparum* infection for treatment decision making, reporting or surveillance, current Pf-LDH-detecting products could have utility until better RDTs become available. The risk–benefit of presumptive treatment of fever *versus* false-negative Pf-LDH RDTs secondary to parasitaemia below 2,000 parasites/µL would need to be carefully considered.

Although the main focus of this study was to assess the performance of Pf-LDH-detecting RDTs, a variety of RDTs that only detect *P. falciparum* using HRP2 were also included. This provided the opportunity to review how these products respond with the a priori assumption that HRP2-detecting RDTs would not detect *pfhrp2*-deleted parasites, an assumption which was confirmed for parasites lacking both *pfhrp2* and *pfhrp3*. The majority of field studies test for the presence of both *pfhrp2* and *pfhrp3* but to date very few have found *pfhrp2*-negative/*pfhrp3*-positive parasites [22]. However, in this study both double- and single-deleted parasites were included, since the structural similarity between HRP2 and HRP3 may provide the opportunity for cross-reactivity [3]. The results demonstrate that some, but not all, HRP2-detecting RDTs return positive results against single-deleted *P. falciparum* at concentrations equivalent to 200 parasites/µL. Hence, it appears that some products tested are able to detect HRP3, as previously reported, and even at lower concentrations [6, 22]. Therefore, the risk of incorrect diagnosis posed by single-deleted mutants is reduced when certain RDT brands are used. In this study, detection of *pfhrp2*-negative/*pfhrp3*-positive parasites for individual HRP2-based RDTs ranged from very limited (1.1%) to almost complete detection (92.0%). This large variation has implications for the detection and surveillance of HRP2-negative parasites, since in some cases only double-deleted parasites will present as RDT-negative, while in other cases both single- and double-deleted parasites will present as RDT-negative. This difference could affect the frequency of false-negative RDT results and also survey estimates of the prevalence of *pfhrp2*-deleted parasites in symptomatic patients, if HRP2-negative RDT results are used as a first screen for mutant parasites that require genotyping [31]. Therefore, buyers should consider these results when selecting an HRP2 RDT to purchase, as the cross-reactivity afforded by HRP3 will reduce the number of false-negative RDT results. For surveillance, estimation of the prevalence of *pfhrp2*-deleted parasites may require inclusion of a sub-set of HRP2-positive RDTs from malaria patients, as well as genotyping for both *pfhrp2* and *pfhrp3*. Furthermore, it is not known if *pfhrp2* single-deleted mutants are a harbinger for *pfhrp3* deletions, and subsequently the double deletions that generate false-negative results on HRP2 test lines.

Combination RDTs that use HRP2 to detect *P. falciparum* and pan-LDH to detect *Plasmodium* spp are widely used in areas where *P. falciparum* and *P. vivax* co-exist. These tests potentially detect HRP2-negative parasites but are likely to misclassify the result as a non-falciparum infection. The results of this study demonstrate that there is large variability in the rate of this type of false positivity between products, a feature that is possibly related to the specific antibody used and dependent on whether the HRP2 band cross-reacts with HRP3, as well as the sensitivity of the pan-LDH test band. The variability in the positivity of the pan-LDH band noted in this study matches that previously reported [32], and suggests reliance on the pan-LDH band to detect HRP2-negative parasites in regions where *P. falciparum* dominates may be unreliable.

An important limitation of this study is the small panel size, particularly when separated into double- and single-deleted samples. Unfortunately, all the single-deleted samples were prepared from two culture lines, as no clinical samples were available. There appears to be no difference in RDT performance against clinical and culture samples in the double-deleted parasites used, suggesting that the use of culture-derived samples has not significantly impacted the results of the study.

## Conclusion

The current study demonstrates that Pf-LDH detecting RDTs respond strongly to high-density *P. falciparum* samples lacking *pfhrp2*, but performance at lower densities is variable. It is recommended that further testing of Pf-LDH detecting RDTs be conducted against a larger and geographically diverse panel of HRP2-negative samples. Surprisingly, many HRP2-detecting RDTs were able to detect single-deleted parasites at low density, a likely positive outcome for clinical management of *P. falciparum* in areas where *pfhrp2*-negative/*pfhrp3*-positive parasites exist, but a potential source of discrepancy for reporting prevalence of HRP2-negative parasites. Ultimately, new targets for *P. falciparum* detection should be explored, especially since optimizing Pf-LDH RDTs has proven difficult for manufacturers, so clinicians and community health workers can have confidence in RDT results to make clear treatment decisions in all malaria endemic regions.

## Data Availability

The data generated or analysed during this study are either included in this published article or available from the World Health Organization publication Malaria rapid diagnostic test performance: results of WHO product testing of malaria RDTs: round 8 (2016-2018) [https://www.who.int/malaria/publications/atoz/9789241514965/en/].

## Abbreviations

CDC: US Centers for Disease Control and Prevention
HRP2: Histidine-rich protein 2
HRP3: Histidine-rich protein 3
Pf: *Plasmodium falciparum*
Pv: *Plasmodium vivax*
Pvom: *Plasmodium vivax, P. ovale, P. malariae*
LDH: Lactate dehydrogenase
RDTs: Rapid diagnostic tests
WHO: World Health Organization
